# Rare presence and function of neuroendocrine cells in the nasal mucosa

**DOI:** 10.3389/fimmu.2024.1394539

**Published:** 2024-08-07

**Authors:** Tine Wils, Wout Backaert, Inge Jacobs, Emma Ruysseveldt, Jonathan Cremer, Ellen Dilissen, Dominique M. Bullens, Karel Talavera, Brecht Steelant, Laura Van Gerven, Katleen Martens, Peter W. Hellings

**Affiliations:** ^1^ KU Leuven Department of Microbiology, Immunology and Transplantation, Allergy and Clinical Immunology Research Group, KU Leuven, Leuven, Belgium; ^2^ Clinical Department of Otorhinolaryngology, Head and Neck Surgery, University Hospitals Leuven, Leuven, Belgium; ^3^ KU Leuven Department of Chronic Diseases and Metabolism, Translational Research Center for Gastrointestinal Diseases, KU Leuven, Leuven, Belgium; ^4^ Department of Pediatrics, University Hospitals Leuven, Leuven, Belgium; ^5^ KU Leuven Department of Cellular and Molecular Medicine, Laboratory of Ion Channel Research Division of Physiology, KU Leuven, Leuven, Belgium; ^6^ KU Leuven Department of Neurosciences, Experimental Otorhinolaryngology Rhinology Research, KU Leuven, Leuven, Belgium; ^7^ University of Antwerp (UAntwerp) Department of Bioscience Engineering, Lab of Applied Microbiology and Biotechnology, University of Antwerp (UAntwerp), Antwerp, Belgium; ^8^ University of Ghent (UGhent) Department of Head and Skin, Upper Airways Research Laboratory, University of Ghent (UGhent), Ghent, Belgium

**Keywords:** neuroendocrine cells, nasal hyperreactivity, nasal mucosa, upper airway epithelium, neurogenic inflammation

## Abstract

There is growing evidence that neurogenic inflammation contributes to the pathophysiology of upper airway diseases, with nasal hyperreactivity (NHR) being a key symptom. The rare neuroendocrine cells (NECs) in the epithelium have been linked to the pathophysiology of bronchial and intestinal hyperreactivity, however their presence in the nasal mucosa and their potential role in NHR remains unclear. Therefore, we studied the presence of NECs in the nasal epithelium of controls, allergic rhinitis patients and chronic rhinosinusitis with nasal polyps patients, and their link to NHR. The expression of typical NECs markers, CHGA, ASCL1 and CGRP, were evaluated on gene and protein level in human samples using real-time quantitative PCR (RT-qPCR), western blot, immunohistochemistry fluorescence staining, RNA scope assay, flow cytometry and single cell RNA-sequencing. Furthermore, the change in peak nasal inspiratory flow after cold dry air provocation and visual analogue scale scores were used to evaluate NHR or disease severity, respectively. Limited gene expression of the NECs markers *CHGA* and *ASCL1* was measured in patients with upper airway diseases and controls. Gene expression of these markers did not correlate with NHR severity nor disease severity. *In vitro*, CHGA and ASCL1 expression was also evaluated in primary nasal epithelial cell cultures from patients with upper airway disease and controls using RT-qPCR and western blot. Both on gene and protein level only limited CHGA and ASCL1 expression was found. Additionally, NECs were studied in nasal biopsies of patients with upper airway diseases and controls using immunohistochemistry fluorescence staining, RNA scope and flow cytometry. Unlike in ileum samples, CHGA could not be detected in nasal biopsies of patients with upper airway diseases and control subjects. Lastly, single cell RNA-sequencing of upper airway tissue could not identify a NEC cluster. In summary, in contrast to the bronchi and gut, there is only limited evidence for the presence of NECs in the nasal mucosa, and without correlation with NHR, thereby questioning the relevance of NECs in upper airway pathology.

## Introduction

The airway epithelium represents the first mucosal barrier protecting the host against infiltration of potential harmful airborne stimuli (1–3). Besides the formation of a physical barrier, the epithelium is equipped with specialized cells and receptors that can detect and respond to noxious stimuli within the inhaled air (4–6). Traditionally, the airway epithelium consists of three major cell types, i.e., ciliated cells, mucus-secreting goblet cells and stem cell-like basal cells (2, 7). With the emerging and growing use of advanced sequencing techniques, the traditional view of the airway epithelium as a sole barrier to the environment has changed. Rather, the airway epithelium is a dynamic cellular structure containing several types of rare specialized cells that are able to respond to environmental changes and interact with the immune and neurogenic system (8–11). One of these rare identified epithelial cells in the lower respiratory epithelium are neuroendocrine cells (NECs) which might play a role in the pathophysiology of neurogenic inflammation (8, 12). Neurogenic inflammation is presumed to be triggered by external non-specific environmental airborne stimuli, such as cigarette smoke, temperature or humidity changes and other irritants, leading to sensory nerve activation with the subsequent release of neuropeptides including substance P and calcitonin gene-related peptide (CGRP) (13). These neuropeptides can directly activate inflammatory cells like mast cells, which leads to the release of classic pro-inflammatory mediators causing inflammation (14). One of the pivotal clinical manifestations of neurogenic inflammation in the upper airways is nasal hyperreactivity (NHR) (15). NHR is defined as the induction of one or more nasal symptoms upon encounter of environmental stimuli, such as cigarette smoke, temperature or humidity changes, strong odors or fragrances, and other irritants (15) It is presumed that NHR is triggered by overactivation of the sensory nerves innervating the respiratory mucosa to non-specific environmental airborne stimuli (1, 15, 16). In physiological conditions, the airways are highly sensitive to airborne stimuli since they are continuously exposed to the environment. Upon encounter with potentially harmful airborne stimuli, the epithelium provides a fast protective response to remove potentially harmful substances from the airways by means of sneezing and rhinorrhea via efferent nerves (17). In case of NHR, there presumably is an overactivation of these sensory neurons, also provoking a response to non-specific environmental airborne stimuli (15, 16). In chronic upper airway diseases, like allergic rhinitis (AR) and chronic rhinosinusitis with nasal polyps (CRSwNP), there is growing evidence that neurogenic inflammation also contributes to the pathophysiology aside from the typical type 2 inflammation (1, 13, 18). The reported prevalence of NHR in patients with upper airway diseases is between 50% and 80%, indicating that neurogenic inflammation is indeed part of the pathophysiology of upper airway diseases (15, 19, 20). The cold dry air provocation test has been shown to be a good tool to objectively quantify NHR in patients with a high sensitivity and specificity in the upper airways (21). As NHR develops upon encounter of airborne stimuli, and NECs are embedded in epithelia, NECs form the prime candidate to be involved in the pathophysiology of NHR (13, 22). Indeed, over the last few years NECs have gained more interest given their possible role in sensory neuron hyperactivation (23–27). NECs act as chemosensors to the external environment that, upon activation, release various neuropeptides like substance P and CGRP. These neuropeptides induce further downstream responses, including activation of sensory neurons located at the base of NECs (8). In the lungs, pulmonary NECs have been shown to respond to changes in oxygen, mechanical stretch and chemical stimuli (23, 28–30). Recently, pulmonary NECs were shown to amplify type 2 allergic responses in mice (23). In the gut, enteroendocrine cells, which display large similarities with pulmonary NECs, were similarly identified as chemosensors that regulate a variety of physiological processes, including gastrointestinal motility and visceral hypersensitivity (24, 25). Pulmonary NECs and their intestinal counterpart, enteroendocrine cells are characterized by expression of Chromogranin A (CHGA) and Achaete-Scute Family bHLH transcription factor 1 (ASCL1) as they both have been shown to be constitutively with the top marker genes of this cell type (8, 12, 31). Chromogranin A (CHGA) is a member of the chromogranin family which belongs to the neuroendocrine secretory proteins, while ASCL1 is a known neurogenic transcription factor with a key role in the development of pulmonary NECs (32–35). Unlike the role of NECs in intestinal and bronchial epithelium, the presence and function of NECs in the nasal epithelium is largely uninvestigated. Therefore, the aim of this study was to evaluate the presence of NECs in the nasal mucosa and their potential role in the induction of NHR in upper airway diseases.

## Materials and methods

### Primary nasal epithelial cell isolation

Primary nasal epithelial cells were isolated from residual surgical waste tissue coming from inferior turbinates of healthy controls and AR patients collected during aesthetic or functional surgery, and from nasal polyp tissue from CRSwNP patients collected during functional endoscopic sinus surgery. Ear-Nose-Throat specialists diagnosed AR patients with symptomatic (≥ 2 nasal symptoms) house dust mite (HDM)-induced AR, and CRSwNP patients based on nasal endoscopy. Control subjects had no clinical signs or history suggestive of AR or CRS. All included subjects were non-smokers (≥ 1 year). Detailed patient’s characteristics can be found in **Supplementary Table 1**. The study was approved by the Ethical Committee Research of University Hospitals Leuven (S65483). Primary nasal epithelial cells were isolated from residual surgical waste tissue as previously reported (4). Collected cells were expanded in bronchial epithelial basal medium supplemented with the SingleQuot Kit (CC-3170, Lonza) or directly seeded on Thincert inserts (Greiner Bio-One) at a concentration of 100,000 – 120,000 cells per Thincert insert using DMEM/F-12 (11320033, Gibco) supplemented with 100 U/mL penicillin, 100 g/mL streptomycin and 2% Ultroser G (15950-017, Sartorius) in liquid-liquid interface. Once a confluent monolayer was formed (approximately 7-10 days), apical medium was removed to allow cell differentiation in the air-liquid interface for an additional 14 days.

### Nasal biopsies

Nasal biopsies were collected from patients with well-document NHR disease status. Patients with HDM-induced AR or CRSwNP, and healthy controls were recruited on a voluntary basis from the outpatient rhinology clinic of the University Hospitals Leuven. All participants were well-characterized by Ear-Nose-Throat specialists and underwent skin prick testing, nasal endoscopy, cold dry air provocation test, and filled in the Visual Analogue Scale (VAS) questionnaire for nasal symptoms. Nasal biopsies were harvested from the inferior turbinates after local anesthesia (cocaine 1%) with a Fokkens forceps and snap frozen in liquid nitrogen. Participants were considered to suffer from NHR using the cold dry air provocation test as previously described (19, 21). Peak Nasal Inspiratory Flow (PNIF) was measured immediately before and after provocation with cold dry air with a PNIF-device (In-Check Nasal Inspiratory Flow Meter, Clement Clarke International, Harlow, UK) and NHR was diagnosed in case of a drop in PNIF of at least 20%. Detailed patient’s characteristics can be found in **Supplementary Table 2**. The study was approved by the Ethical Committee Research of University Hospitals Leuven (S63139).

### Ileum biopsies

Ileum samples were collected from the most proximal unaffected regions of colon rectal cancer and Crohn’s disease patients. Detailed patient’s characteristics can be found in **Supplementary Table 3**. The study was approved by the Ethical Committee Research of University Hospitals Leuven (S53684, S68720). Biopsies were put in RLT lysis buffer (79216, Qiagen) for RT-qPCR experiments, in PBS containing 5% BSA for western blot experiments or in cold RPMI medium for flow cytometry experiments.

### Western blot

Cells lysates were harvested in cold NP-40 lysis buffer (50 mM Tris.HCl (pH 8.0), 150 mM NaCl, 1% NP-40) containing 1X cOmplete Protease Inhibitor (CO-RO, Roche) and 0.4 mM PMSF protease inhibitor in dry isopropanol (36978, Thermo Fisher Scientific). Protein was extracted and total protein concentration was determined using the Pierce BCA protein assay kit (23227, ThermoFisher Scientific). Protein samples were resuspended in LDS Sample Buffer (1X) (NP007, Invitrogen) and Reducing Agent (1X) (NP0009, Invitrogen), after which denatured proteins were separated by electrophoresis using SDS-PAGE gels (NP0323, Invitrogen). Separated proteins were electroblotted onto an activated PVDF membrane (10600023, VWR International) in transfer buffer (31.2 mM Tris-Base, 0.24 M glycine, 20% methanol, 2% antioxidant). Membranes were blocked with 5% milk powder (MP) (5% milk, 20 mM Tris-HCl, pH 7.6), followed by overnight incubation at 4°C with primary antibodies anti-Chromogranin A antibody (1:1,000; ab45179, abcam), anti-ASCL1 polyclonal antibody (1:1,000; PA5-77868, invitrogen) or anti- Glyceraldehyde-3-Phosphate Dehydrogenase (GAPDH) monoclonal antibody (6C5) (1:10,000; AM4300, Invitrogen) in 1% MP. Primary antibodies were detected via enhanced chemiluminescence (NEL105, PerkinElmer) using HRP-linked secondary antibodies (anti-rabbit; 1:5,000; 32460, Thermo Scientific or anti-mouse; 1:5,000; 32430, Thermo Scientific). The signal was visualized with the ImageQuant LAS500 (Bioké, Leiden, The Netherlands).

### Real-time quantitative polymerase reaction

Biopsies were homogenized in RLT lysis buffer (79216, Qiagen) using Lysing Matrix D and a FastPrep-24-device (116913100 and 116004500, MP Biomedicals) and RNA was extracted using the RNeasy Mini Kit (74106, Qiagen). cDNA was obtained using a High-Capacity cDNA Reverse Transcription kit (4368814, Thermo Fisher Scientific) starting from 2 µg RNA. RT-qPCR was performed for genes of interest *CHGA* and *ASCL1*, and for housekeeping genes actin beta (*ACTB*) and receptor for activated C kinase 1 (*RACK1*) (sequences in **Supplementary Table 4**) with the CFX Connect Real-Time PCR Detection System (Bio-rad, Hercules, California, USA). cDNA plasmid standards of each gene were used to quantify the amount of target genes in unknown samples.

### Immunohistochemistry fluorescence staining

Paraffin-embedded tissue slides (5 μm) of nasal and ileum biopsies were subjected to antigen retrieval in citrate buffer (pH 6). Proteins of interest were detected using the following primary antibodies: anti-chromogranin A antibody (1:100, ab45179, abcam), anti-chromogranin A antibody (LK2H10) (1:100, MA5-13096, Invitrogen), anti-CGRP antibody (1:50, ABS 026-04-02, Invitrogen), anti-KRT5-Alexa Fluor 488 antibody (1:100, ab193894, abcam), anti-MUC5AC antibody (45M1) (1:150, MA5-12178, Invitrogen) and anti- acetylated tubulin antibody (clone 6-11B-1) (1:300, T7451, Sigma-aldrich). Secondary antibodies were anti-rabbit AlexaFluor 594 (1:500, A21207, ThermoFisher) and anti-mouse AlexaFluor 488 (1:500, A21202, ThermoFisher). Lastly, nuclei were stained with 4′-6-diamidino-phenylindole dihydrochloride–containing (3nM, D9542, Sigma-Aldrich). After staining, tissues were mounted and images were taken using the ZEISS axioscan 7 (Zeiss, Oberkochen, Germany).

### RNA scope

Paraffin-embedded nasal and ileum biopsy slides (5 μm) were prepped and stained using the RNA scope 2.5 reagent Kit (322350, Advanced Cell Diagnostics). Briefly, paraffin-embedded samples were baked for 1h at 60°C and deparaffinized in 100% xylene and 100% ethanol. Airdried slides were pretreated with RNA scope hydrogen peroxide (322330, Advanced Cell Diagnostics) for 10 min, followed by antigen retrieval (322000, Advanced Cell Diagnostics) for 15 min at 99°C. Slides were incubated with Protease plus reagents (322330, Advanced Cell Diagnostics) for 30 min in the humidity and temperature controlled HybEZ oven. The anti-CHGA RNAscope Probe - Hs-CHGA (311111, Advanced Cell Diagnostics) was hybridized for 2h at 40°C. Signal amplification was obtained by sequential hybridization of 6 amplifiers, whereafter signal detection was performed using the Fast RED working solution (322360, Advanced Cell Diagnostics). Slides were counterstained with Hematoxylin, after which mounted slides were evaluated using the ZEISS axioscan 7 (Zeiss, Oberkochen, Germany).

### Single cell RNA-sequencing

Single cell suspensions of surgically collected inferior turbinates were obtained by enzymatic digestion using liberase (87.5 µg/ml, 5401127001, Roche) and DNase I (300 µg/ml, 10104159001, Roche) in RPMI with 10% FBS for 30 min with additional mechanical disruption using a 16G needle after both 15 min and 30 min of enzymatic digestion. Digestion was stopped by adding EDTA (20 mM, 15575020, Invitrogen), whereafter red blood cells were lysed using 9.9% NaCl solution. Negative selection for immune cells, myeloid cells and fibroblasts was performed using anti-CD45 (11153D, Invitrogen), anti-CD15 (11137D, Invitrogen) and anti-CD31 (11155D, Invitrogen) dynabeads, respectively. Filtered cells (70 μm, 542070, Greiner) were loaded on the 10X Genomics Chromium 3’ device (3’ V2, 10X Genomics, Pleasanton, California, USA), and afterwards RNA libraries were constructed and sequenced using the NovaSeq 6000 S4 Reagent Kit V1.5 (20028312, Illumina) and using the NovaSeq 6000 device (Illumina, San Diego, California, USA). Raw sequencing files were cleaned, mapped to the human reference genome, filtered, normalized, demultiplexed and counted using fastaq-mcf, fastQC, Cell Ranger and fastMnM, after which data was analyzed using different R tools, such as Seurat, Harmony and Bioconductor in R 4.2.1 (36–41).

### Single cell staining and flow cytometry

Single cells solutions of nasal tissues were obtained by overnight enzymatic digestion of surgically collected tissue in 1 mg/ml protease XIV (P5147-1G, Sigma) in Dulbecco’s modified Eagle’s medium (DMEM/F12; Lonza BioWhittaker) supplemented with 100 U/mL penicillin, 100 g/mL streptomycin and 2% Ultroser G (15950-017, Sartorius). Surgically obtained ileum tissue was mechanically disrupted followed by enzymatically digestion for 40 min at 37°C and 5% CO_2_ in pre-heated HBSS Ca^+^Mg^+^ buffer containing 1 mg/ml collagenase D (COLLD-RO, Roche), 20 units/ml DNase I (EN0521, Thermo Fisher Scientific) and 0.02 mM HEPES (15630080, Gibco). 100,000 to 1,000,000 filtered (70 μm, 542070, Greiner) single cells were collected and incubated for 25 min with 1 µl/ml eBioscience Fixable Viability Dye eFluor 780 (65-0865-14, Invitrogen). Cells were blocked using 5 µl/ml human serum for 10 min at 4°C, after which cells were stained with 50 µl/ml Brilliant Violet 650 anti-human CD326 (EpCAM) Antibody (BV650, Biolegend) for 30 min at 4°C. Sequentially, intracellular staining was performed using Flow Cytometry Fixation & permeabilization Buffer Kit I (FC009, Biotechne) with 50 µl/ml PE Anti-Human Chromogranin A Clone S21-537 (RUO) antibody (5564563, BD Pharmingen). Fixated cells were acquired using the BD LSRFortessa Cell Analyzer (BD biosciences, New Jersey, USA) equipped with FACSDiva software. Flow cytometric data was analyzed in FlowJo v10.7.1 (BD Biosciences, New Jersey, USA). Analysis included removal of debris, doublet cells and dead cells.

### Statistics

Data was analyzed using GraphPad Prism 10 (GraphPad Software, La Jolla, Calif). Differences between two groups were analyzed by the Mann-Whitney U test. Differences between multiple groups were analyzed by the One-way ANOVA or the Kruskal-Wallis test with *post hoc* analysis, depending on normality distribution. Categorical values were compared by using the Chi square test and correlations were assessed by using the Spearman r test. Values were considered significantly different at a p value of less than 0.05 adjusted to the appropriate multiple testing correction (Bonferroni) if needed.

## Results

### Limited expression of NECs markers in the nasal mucosa of patients with upper airway disease and healthy subjects

**Figure 1:**
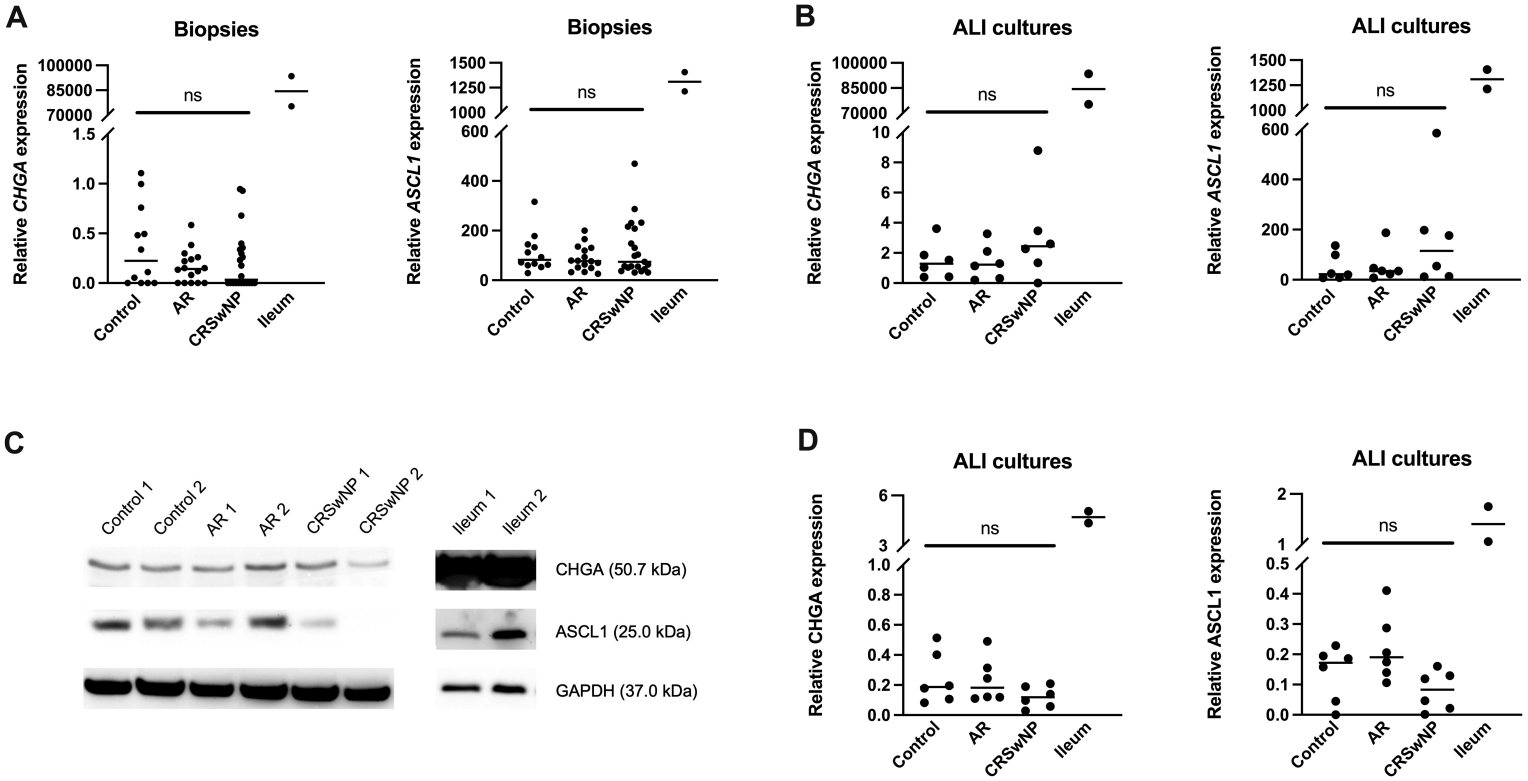
Expression of the NEC markers CHGA and ASCL1 in nasal biopsies and cultured primary nasal epithelial cells. **(A)** *CHGA* and *ASCL1* mRNA expression levels in nasal biopsies and ileum biopsies relative to the expression of the housekeeping genes *ACTB* and *RACK1*. **(B)** *CHGA* and *ASCL1* mRNA expression levels in air-liquid interface cultured primary nasal epithelial cells and ileum samples relative to the expression of the housekeeping genes *β-actin* and *RACK1*. **(C)** Representative western blot of CHGA, ASCL1 and GAPDH (housekeeping protein) protein expression levels in cultured primary nasal epithelial cells and ileum samples. **(D)** Quantification of CHGA and ASCL1 protein expression levels relative to GAPDH expression levels based on western blot analysis. AR, allergic rhinithis; CRSwNP, chronic rhinosinusitis with nasal polyps; CHGA, chromogranin A; ASCL1, Achaete-Scute Family bHLH transcription factor 1; data represented as individual values with median, ns = p > 0.05.

**Figure 2:**
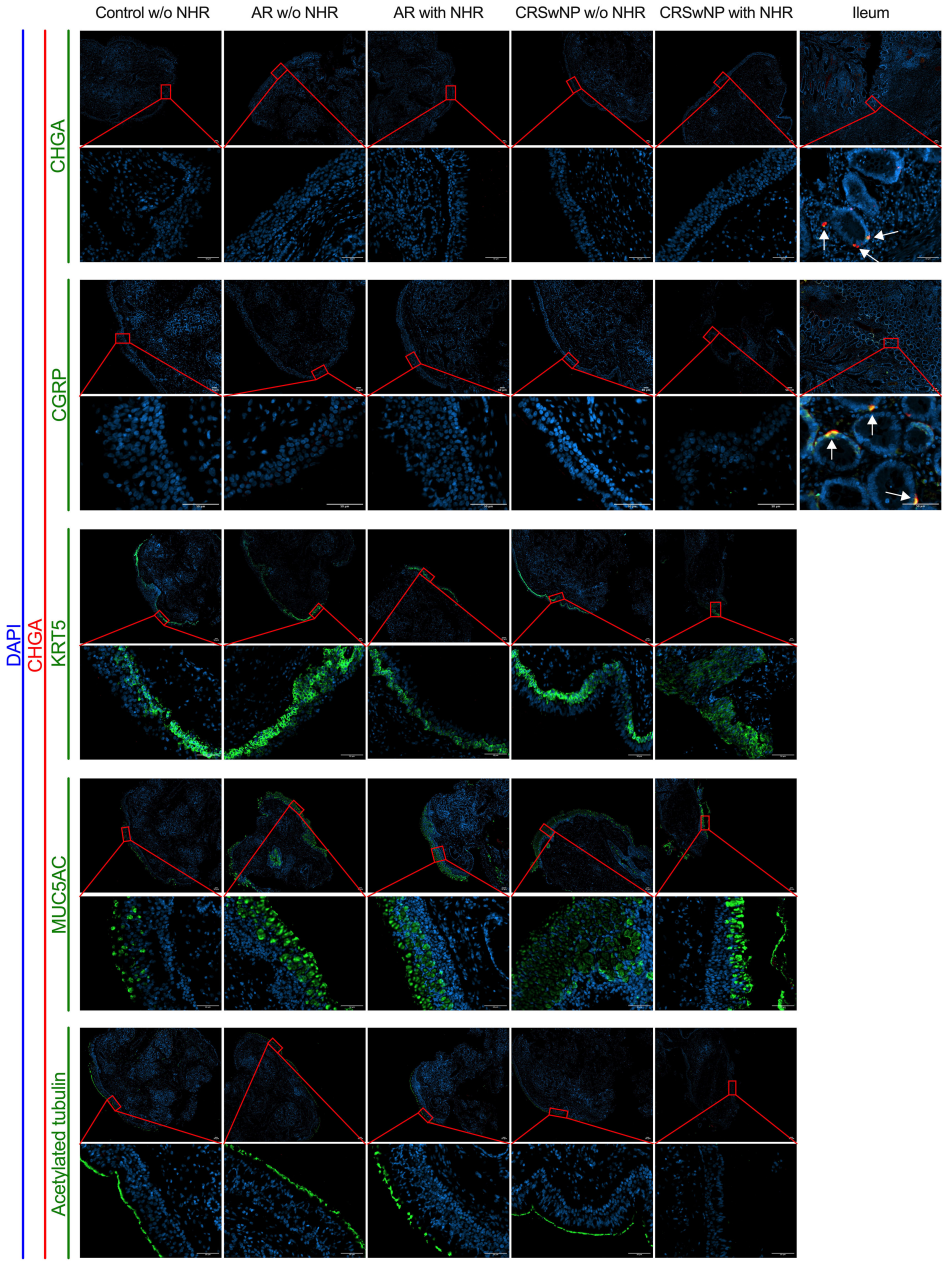
Immunohistochemistry fluorescence staining of neuroendocrine, basal, goblet and ciliated epithelial cells in nasal and ileum biopsies of patients and controls. Representative immunohistochemistry fluorescence staining of the neuroendocrine and entroendocrine cell makers: CHGA (red)/CHGA (green) and CHGA (red)/CGRP (green) in both nasal biopsies from controls without NHR, AR with and without NHR patients and CRSwNP with and without NHR patients and ileum biopsies. Representative immunohistochemistry fluorescence staining of basal cells (KRT5, green), goblet cells (MUC5AC, green) and ciliated cells (acetylated tubulin, green) in combination with CHGA (red) in nasal biopsies from controls without NHR, AR with and without NHR patients and CRSwNP with and without NHR patients. All stainings were costained with DAPI (blue) to visualize the nuclei. NHR = nasal hyperreactivity; AR, allergic rhinithis; CRSwNP, chronic rhinosinusitis with nasal polyps; CHGA, chromogranin A; CGRP, calcitonin gene-related peptide; KRT5, Keratin 5; MUC5AC, Mucin-5AC, DAPI = 4′,6-diamidino-2-phenylindole; scale bar = 50 µm.

**Figure 3:**
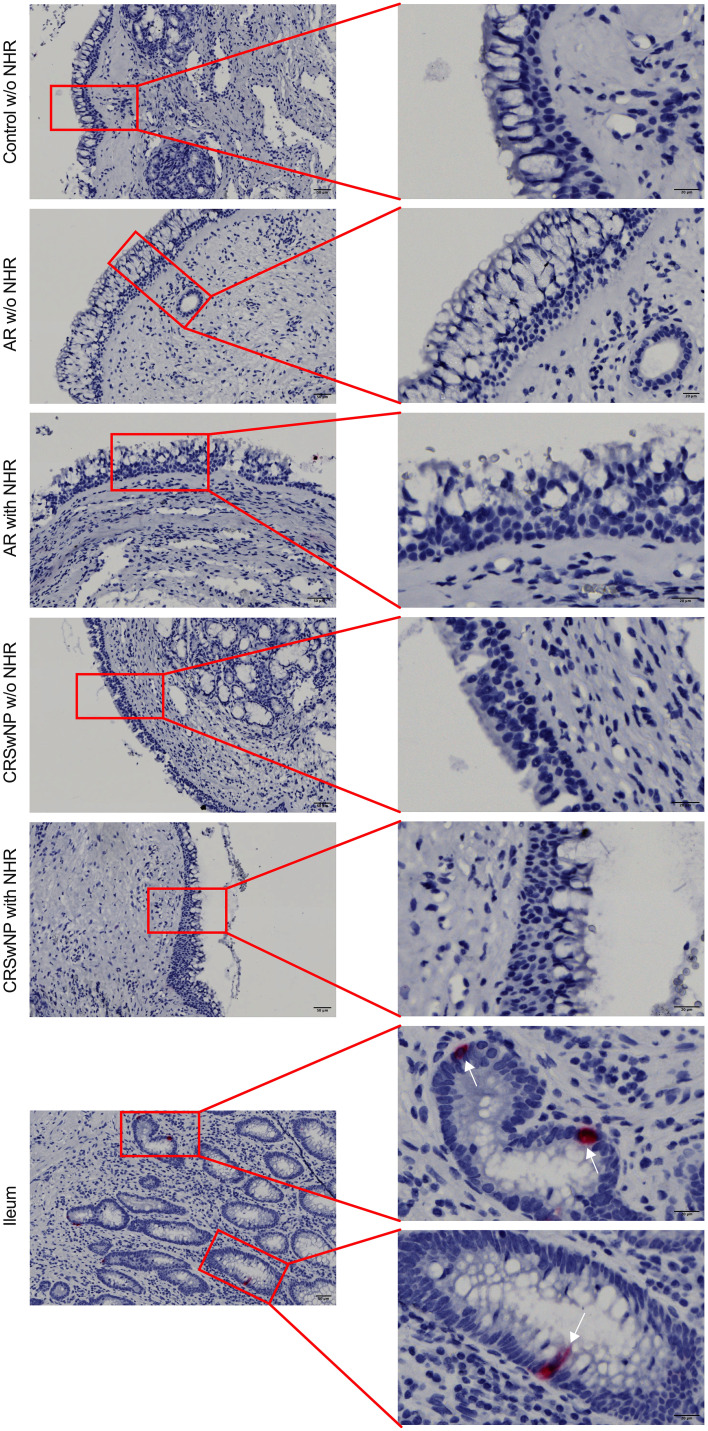
Detection of *CHGA* RNA molecules in ileum and nasal biopsies using RNA scope RED assay. Representative *CHGA* RNA detection in nasal biopsies from controls without NHR, AR with and without NHR patients and CRSwNP with and without NHR patients and ileum samples as determined by the RNA scope RED assay. NHR, nasal hyperreactivity; AR, allergic rhinithis; CRSwNP, chronic rhinosinusitis with nasal polyps; CHGA, chromogranin A; scale bar left = 50 µm; scale bar right = 20 µm.

**Figure 4:**
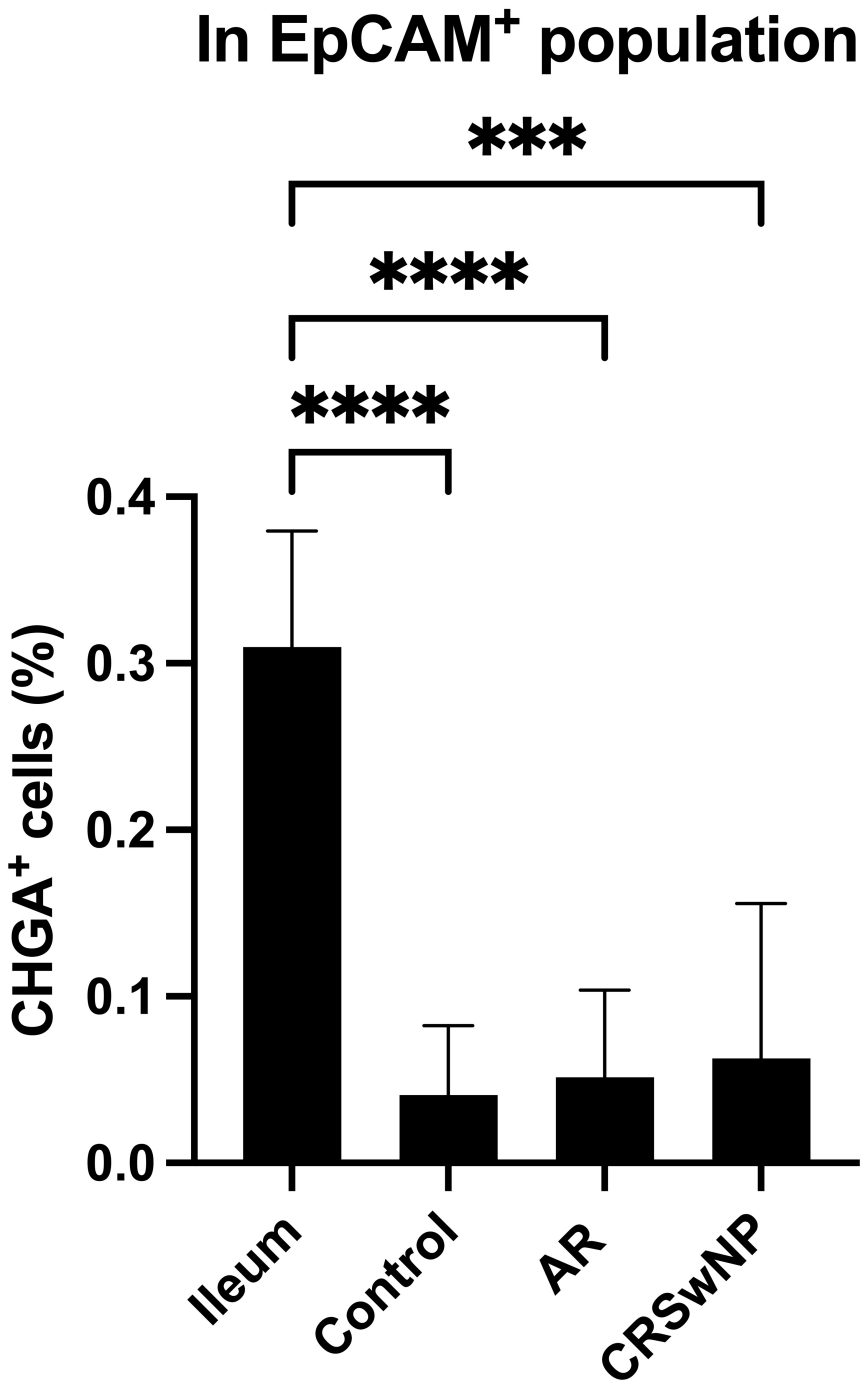
Quantification of CHGA^+^ cells in ileum and nasal biopsies as determined by flow cytometry. Percentage of CHGA^+^ cells within the epithelial cell population defined as EpCAM^+^ expression in ileum tissue and nasal tissue from controls, AR patients and CRSwNP patients (n = 5/group). CHGA, chromogranin A; EpCAM: epithelial cellular adhesion molecule; AR, allergic rhinithis; CRSwNP, chronic rhinosinusitis with nasal polyps; data represented as mean ± SD, *** p < 0.005; **** p < 0.001.

To investigate whether NECs are present in the nasal mucosa of patients and controls, the expression of NEC markers CHGA and ASCL1 in nasal biopsies and in primary nasal epithelial cell cultures was evaluated. Unlike clear expression of *CHGA* and *ASCL1* in ileum samples, limited *CHGA* and *ASCL1* expression was measured in nasal biopsies and primary nasal epithelial cells (**Figures 1A, B**). No significant differences in *CHGA* and *ASCL1* expression levels were found between controls, AR patients and CRSwNP patients (**Figures 1A, B**). The limited mRNA expression of *CHGA* and *ASCL1* in primary nasal epithelial cell cultures was confirmed on protein level, while ileum samples clearly showed more CHGA and ASCL1 protein expression (**Figures 1C, D**). No significant difference in protein expression was found between controls, AR patients and CRSwNP patients (**Figures 1C, D**). This observation was confirmed with immunohistochemistry fluorescence staining. A clear double positive (CHGA^+^/CHGA^+^ and CHGA^+^/CGRP^+^) cell population was observed in ileum samples, while this double positive population was absent in nasal biopsies, irrespective of disease or NHR status (**Figure 2**). In contrast, immunohistochemistry fluorescence staining of the three main cell types in the upper airway epithelium being KRT5 for basal cells, MUC5AC for goblet cells and acetylated tubulin for ciliated cells, could be detected. These results were further confirmed using the RNA scope assay which detected *CHGA* ^+^ cells in the ileum, while no *CHGA^+^* cells were detected in controls without NHR, AR with and without NHR and CRSwNP with and without NHR (**Figure 3**). With flow cytometry, significantly more CHGA^+^ cells were detected in the epithelial cellular adhesion molecule (EpCAM)-positive epithelial cell population in ileum samples compared to control, AR and CRSwNP samples (**Figure 4**; **Supplementary Figure 1**).

**Figure 5:**
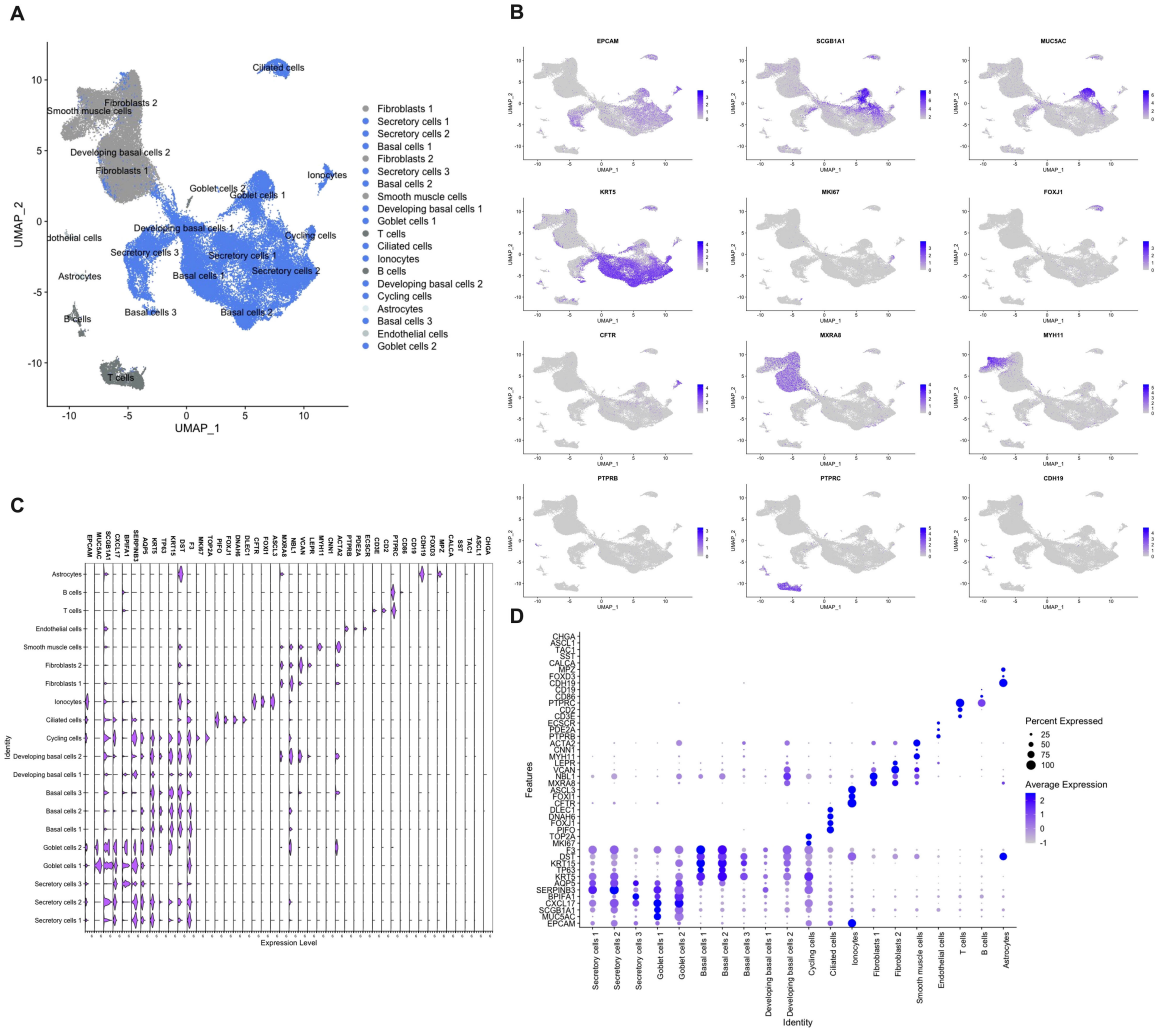
Single cell RNA-sequencing of cells isolated from nasal biopsies from patients and controls. **(A)** Umap clustering and annotation of single cell RNA-sequencing of allergic rhinitis patients (n=2) and controls (n=2) coloured based on epithelial (blue) and non-epithelial (grey) cells. **(B)** Feature plots of top marker genes for each cell cluster. **(C)** Violin plot showing gene expression of cluster and NEC markers. **(D)** Dot plot displaying mRNA expression levels of cluster and NEC markers.

As NECs are a rare cell population (< 0.5%), and potentially are missed with less sensitive methods, we used single cell RNA-sequencing to define the presence of NECs in the nasal mucosa (8, 11, 12). In total 73,487 cells from inferior turbinates of four well characterized subjects of which two controls and two HDM-induced AR patients were sequenced. After quality control and filtering, 61,655 cells remained with a high quality of sequencing data (**Supplementary Figure 2**). After Harmony integration to remove batch effects, unsupervised graph-based clustering was performed. In total, 20 clusters were defined of which 13 were identified as epithelial cell clusters (blue) after annotation (**Figure 5A**). Non-epithelial cell clusters included fibroblasts characterized by *MXRA8, NBL1, VCAN* and *LEPR* expression and smooth muscle cells characterized by *MYH11, CNN1* and *ACTA2* expression (**Figures 5B–D**). Other smaller non-epithelial cell clusters were identified as endothelial cells characterized by *PTPRB, PDE2A* and *ECSCR* expression, as well as astrocytes which were identified by expression of *CDH19, FOXD3* and *MPZ* (**Figures 5B–D**). Lastly, the non-epithelial cell clusters contained two immune cell clusters, being T and B cells, characterized by expression of *CD3E, CD2, PTPRC* and *CD86, CD19*, respectively (**Figures 5B–D**).

Within the epithelial cell clusters, most cells were identified as secretory cells, including goblet cells. Secretory and goblet cells were identified by the expression of general secretory markers such as *SCGB1A1, CXCL17, BPIFA1, SERPINB3* and *AQP5* (**Figures 5B–D**). Despite strong transcriptional similarity, goblet cells were identified by having higher *MUC5AC* expression which is the best-known marker for goblet cells in the upper airways (**Figures 5B–D**). The second largest population that was identified were the basal cells. In total, three basal cell populations and two developing basal cell populations were identified. All basal cell clusters expressed *KRT5, TP63, KRT15, DST* and *F3* to a different extend (**Figures 5B–D**). More stem cell like basal cell clusters (basal cells 1 and 2) expressed more *KRT5* and *TP63*, while the more developing basal cells expressed less *KRT5* and *TP63* and started to show low expression of the beforementioned secretory genes (**Figures 5B–D**). Cycling cells were identified by their mixed expression of both secretory and basal genes, as well as by added expression of genes involved in cell cycling such as *MKI67* and *TOP2A* (**Figures 5B–D**). In our dataset, a lower amount than expected of ciliated cells, characterized by the expression of *PIFO, FOXJ1, DNAH6* and *DLEC1*, were sequenced. (**Figures 5B–D**). Furthermore, also a cell cluster of the rare cell type, the ionocytes, defined by *CFTR, FOXI1* and *ASCL3* expression, was identified (**Figures 5B–D**). In contrast, known marker genes of NECs, such as *CHGA, ASCL1, TAC1, SST* and *CALCA*, could not be found to identify a single cell population of NECs in our dataset (**Figures 5C, D**). This in contrast with the analysis of the existing CZ CELLxGENE Discover database which shows uniform expression of *CHGA* in NECs across different human tissues (**Supplementary Figure 3**) (42). Most pulmonary NECs also express *ASCL1* and *CALCA*, and to a lesser extent *SST* and *TAC1* (**Supplementary Figure 3**) (42). In conclusion, with the use of several techniques, we were not able to detect the presence of NECs in the nasal mucosa of patients with upper airway disease nor in healthy subjects.

### No correlation of NECs marker expression with nasal hyperreactivity in upper airway disease

**Figure 6:**
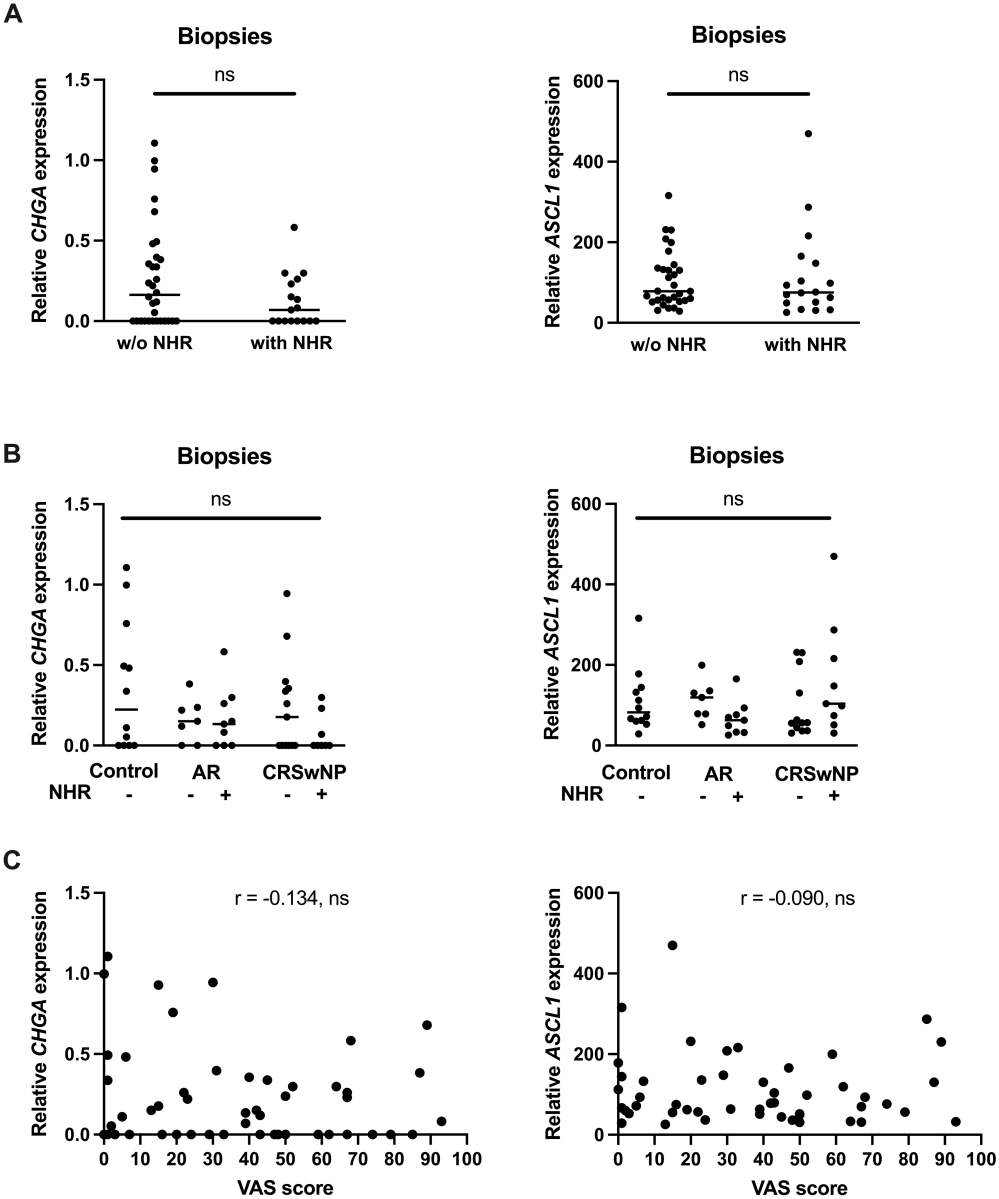
Correlation between the expression of the NEC markers *CHGA* and *ASCL1* and nasal hyperreactivity in upper airway disease. **(A)** mRNA expression levels of *CHGA* and *ASCL1* in human nasal biopsies with and without NHR. **(B)** mRNA expression levels of *CHGA* and *ASCL1* in human nasal biopsies in controls, AR with and without NHR patients and CRSwNP with and without NHR patients. **(C)** Correlation of *CHGA* and *ASCL1* expression with the VAS score of total nasal symptoms as measure of disease severity. NHR, nasal hyperreactivity; AR, allergic rhinithis; CRSwNP, chronic rhinosinusitis with nasal polyps; VAS, visual analogue score; CHGA, chromogranin A; ASCL1, Achaete-Scute Family bHLH transcription factor 1; data represented as individual values with median, ns = p > 0.05.

Since NHR is a hallmark of neurogenic inflammation, we wanted to know if the limited expression levels of *CHGA* and *ASCL1* were correlated with the presence of NHR in patients. Subjects were considered to have NHR if they had a drop of at least 20% in their PNIF after cold dry air exposure as an objective measurement for NHR. No significant differences in *CHGA* and *ASCL1* expression could be found in subjects without NHR compared to patients with NHR (**Figure 6A**). When the subjects were further classified based on their upper airway disease, being controls, AR patients and CRSwNP patients, as well as based on their NHR diagnosis, we also could not observe a significant difference in *CHGA* or *ASCL1* expression levels (**Figure 6B**). Furthermore, no significant correlation between the expression of *CHGA* or *ASCL1* with the VAS score of total nasal symptoms, or other measurements of disease severity, could be detected (**Figure 6C**; **Supplementary Figures 4**, **5**).

## Discussion

There is increasing evidence that NECs play a possible role in sensory neuron hyperactivation (23–26). The role of NEC-like cells in the pathology of upper airways diseases has not been thoroughly studied yet. An extensive analysis of the presence and potential involvement of NECs in NHR in the upper airways of patients with upper airway disease and control subjects was performed. Surprisingly, only limited expression of NECs markers on both RNA and protein level in the upper airway epithelium was found, irrespective of disease status, using pooled methods in which we could not specify from which cells these signals were derived from. In single cell methods, no evidence of NECs in the upper airways was found. Western blotting detects pooled protein signals from all cells in a sample but cannot specify the source cells, while flow cytometry quantifies protein expression in individual cells, providing cell-specific analysis. The strong CHGA response in the ileum by western blotting, compared to only 0.5% detection of CHGA^+^ cells by flow cytometry, highlights the differences between these techniques in sensitivity and specificity. Flow cytometry clearly detects single CHGA^+^ cells in the ileum, however, in the nasal EpCAM^+^ population, the CHGA^+^ cell population and expression are so minimal that it is challenging to definitively confirm the presence of CHGA^+^ cells within the nasal epithelial cell population, thereby questioning the presence of neuroendocrine cells in the nasal epithelium.

Chromogranins, such as CHGA, belong to the family of proteins that constitute a major component of the secretory granules of various endocrine and neuroendocrine cells and is therefore considered as a general marker for NECs (43). In daily clinical setting, CHGA is a well-established biomarker to diagnose neuroendocrine tumors (44). Furthermore, *CHGA* was consistently identified as a top marker gene for NECs in both the lungs and gut (8, 12, 45, 46). Surprisingly, *CHGA* and other NEC markers could not be observed in our dataset. Only four subjects were sequenced, which might be too limited to identify NECs. Nevertheless, we have sequenced up to 20,000 cells per subject, which, based on the estimated prevalence, should have resulted in 200 to 1,000 sequenced NECs. It might be plausible that NECs in the upper airways are characterized by other unidentified marker genes. For this reason, CHGA detection was complemented with the additional marker ASCL1. *Ascl1* knock out studies demonstrated that *Ascl1* is not only an early expressed marker of pulmonary NECs, but is also necessary but not sufficient for pulmonary NECs cell fate specification (23, 47, 48). In addition, CHGA detection was further complemented with the detection of neuropeptides associated with neurogenic inflammation like CGRP, TAC1 and SST (11, 12, 23). In mice, CGRP was shown to be expressed in 95% of pulmonary NECs, and was shown to be released by pulmonary NECs upon allergen challenge, thereby highlighting the role of CGRP as a classical NEC neuropeptide (23, 49). Despite the combination of CHGA detection with multiple other known NEC-associated markers, no relevant expression in the nasal mucosa could be found. Although *CHGA*, *ASCL1* and *CALCA* (encoding CGRP) are all considered as canonical NECs marker, it is still plausible that nasal NECs are characterized by other markers such as *GRP*, *PIEZO2* or *NMU* (42, 49, 50).

Pulmonary NECs first differentiate as scattered solitary cells, whereafter they migrate to form clusters called neuroepithelial bodies (NEB) (30, 51). NEB are frequently observed at the bifurcation points of branching airways (52, 53). This specific location at the airway branch points is also the location were inhaled aerosolized particles congregate, therefore positioning the pulmonary NECs at the ideal location to detect and respond to inhaled environmental particles (54). Additionally, in the lower airways, pulmonary NECs have been described as intrapulmonary chemosensors to detect several environmental stimuli such as oxygen, nicotine, mechanical changes, and succinate (55–64). In the intestine, enteroendocrine cells respond to irritants, metabolites, norepinephrine or interleukin (IL)-33 via activation of TRPA1, olfactory receptor 558, alpha-2A adrenergic receptor and IL-33 receptor, respectively (24, 65). Activation of the enteroendocrine cells through one of the aforementioned receptors results in the release of 5-HT leading to activation of the nearby serotonergic afferent nerve endings (24, 65). As mentioned earlier, a variety of intestinal physiological processes, including gastrointestinal motility and visceral hypersensitivity have been shown to be regulated via 5-HT release (24, 25, 65). Given the accumulation of pulmonary NECs at airway branch points and the chemosensory function of NECs in the lower airways and intestine, it can be assumed that if NECs are present in the nasal epithelium, they would be localized at regions with the highest possibility to encounter environmental triggers. In the nasal cavity, the inferior turbinate represents the best hypothetical location for NECs (66). All samples used in this study were therefore coming from inferior turbinates of controls and AR patients. However, we cannot fully exclude that NECs are localized at a different location in the nasal cavity.

Pulmonary NECs and their intestinal counterpart are known to be extremely rare cell types, with their prevalence being estimated to be less than 0.5% of the epithelium (45–47). It is possible that, even with very sensitive techniques, NECs could not be detected in the upper airway epithelium due to their scarcity. Unlike previous publications which successfully identified NEC clusters in intestines and in the lower airways, we could not detect any NECs in the nasal mucosa using single cell RNA-sequencing (12, 42, 45, 67). When applying all the other assays to ileum samples, enteroendocrine cells were successfully detected, emphasizing that NECs are unlikely to be present in nasal mucosa. The lack of NECs being present in the upper airways was unexpected, given the implication of pulmonary NECs in several pulmonary diseases. Increased numbers of pulmonary NECs were observed in lungs of asthmatic patients (23). Pulmonary NECs play a role in amplifying type 2 inflammation and goblet cell hyperplasia in an allergen-induced mouse model via CGRP and GABA release, respectively (23). Also, chronic obstructive pulmonary disease (COPD) patients have a higher number of pulmonary NECs and pulmonary NECs products in their airways compared to healthy controls (68). Both in COPD patients and in smokers, the major risk factor of COPD, higher levels of bombesin-like peptide were found, suggesting an association with pulmonary NECs hyperplasia and increased sensitivity to chemical stimuli possibly leading to COPD development (69). In neuroendocrine cell hyperplasia of infant syndrome it was shown that patients showed NEC hyperplasia mainly in the distal respiratory airways (70, 71). Furthermore, NECs have also been thought to be the originating cancer cells in neuroendocrine small cell lung cancer (72, 73).

Despite the association of NECs in lower airway diseases, no correlation was found between NEC marker expression and disease or NHR severity in the upper airways. Therefore, it is unlikely that increased numbers of NECs in the upper airways are responsible for upper airway diseases or NHR. As mentioned, the extremely rare nature of NECs, as well as their scattered location can hamper the detection of a relevant correlation. Furthermore, the functional capacity of NECs was not investigated. Different studies have already used specific ASCL1-cre-mediated knock out or labeling of NECs in the murine lower airways to investigate the functional role of NECs (23, 49, 64, 74, 75). An analogue upper airway epithelial specific NEC knock in or knock out mouse model could be used to elucidate the potential functional role of NECs in the upper airways.

In conclusion, only limited expression of NEC markers on both gene and protein level was found in the human upper airway epithelium using multiple methods in which we could not specify from which cells these signals were derived from. Furthermore, no correlation between NECs and NHR could be detected. In contrast, enteroendocrine cells were detected in the ileum using the same protocols. In our single cell RNA-sequencing dataset, no NECs cluster or any type of cells in the nasal mucosa expressing NEC-associated markers was detected. Therefore, the implication of NECs in upper respiratory diseases remains questionable. This in contrast with the bronchial and intestinal epithelium where NECs have been described to be present, to act as chemosensor to environmental stimuli and are associated with various pathophysiology’s.

## Data availability statement

The datasets presented in this study can be found in online repositories. The names of therepository/repositories and accession number(s) can be found below: https://www.ncbi.nlm.nih.gov/geo/, GSE261706.

## Ethics statement

The studies involving humans were approved by Ethical Committee Research of University Hospitals Leuven. The studies were conducted in accordance with the local legislation and institutional requirements. The participants provided their written informed consent to participate in this study.

## Author contributions

TW: Conceptualization, Data curation, Funding acquisition, Investigation, Methodology, Visualization, Writing – original draft, Writing – review & editing. WB: Data curation, Methodology, Writing – review & editing. IJ: Methodology, Writing – review & editing. ER: Methodology, Writing – review & editing. JC: Methodology, Writing – review & editing. ED: Methodology, Writing – review & editing. DB: Supervision, Writing – review & editing. KT: Supervision, Writing – review & editing. BS: Conceptualization, Data curation, Methodology, Supervision, Writing – review & editing. LV: Methodology, Supervision, Writing – review & editing. KM: Methodology, Supervision, Writing – review & editing. PH: Supervision, Writing – review & editing, Funding acquisition, Investigation, Methodology, Validation, Conceptualization.
