# Supplementary material for: Rare presence and function of neuroendocrine cells in the nasal mucosa
Source: Front Immunol. 2024 Aug 7;15:1394539. doi: 10.3389/fimmu.2024.1394539 (PMC11339793; doi:10.3389/fimmu.2024.1394539)
Supplement: Supplementary file 1 [file DataSheet_1.pdf]

Supplementary Materials

Supplementary Figures and Tables

1.1 Supplementary Figures

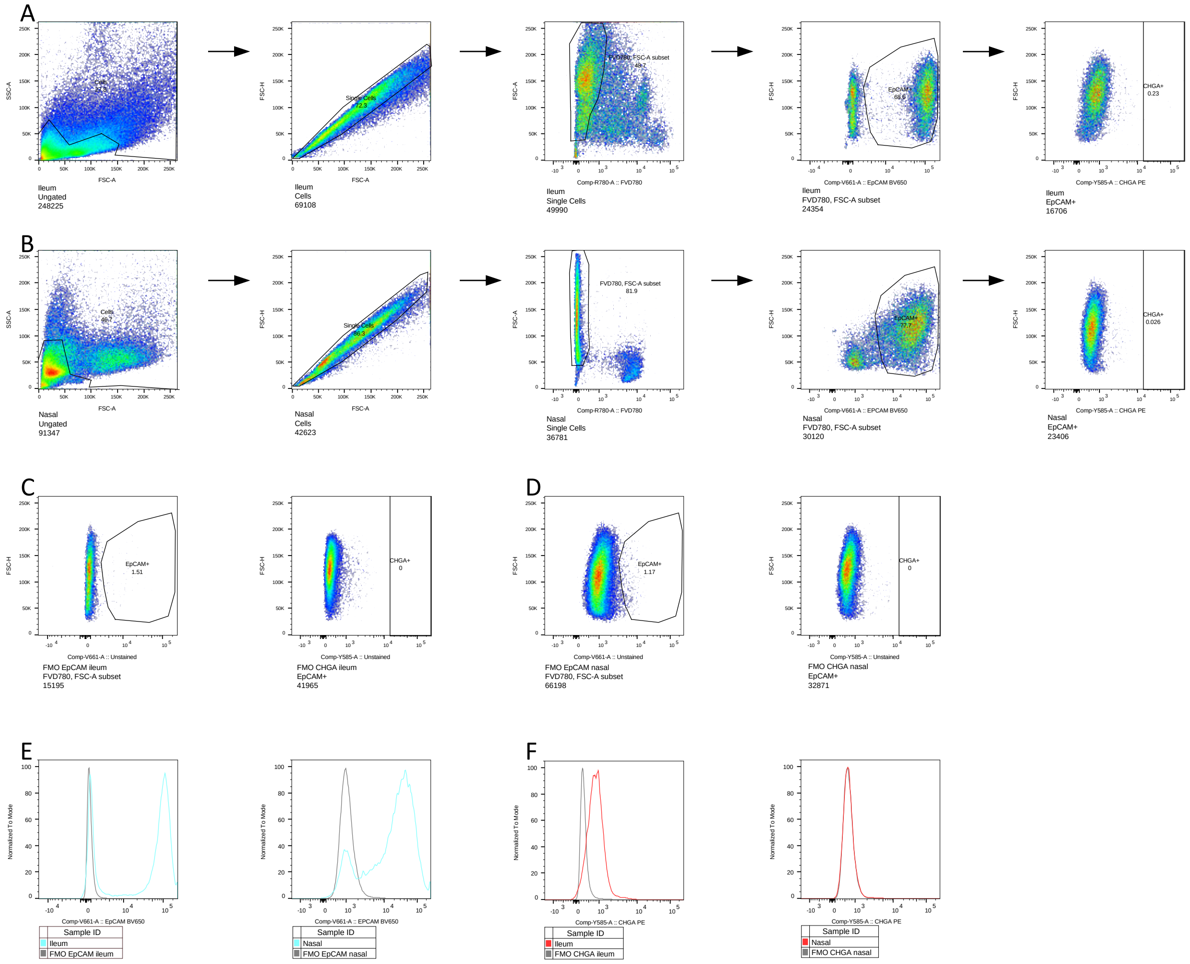

Supplementary figure 1: Gating strategy of EpCAM- and CHGA-positive cells detected by flow cytometry. Neuroendocrine cells were identified via subsequently gating of cells, single cells and viable cells (FVD780$^-$). On the viable cell population, epithelial cells were identified as EpCAM$^+$ cells in which CHGA$^+$ cells were identified as neuroendocrine cells. Representative gaiting strategies for ileum tissue **(A)** and all nasal tissues, being control tissue, AR patient tissue and CRSwNP patient tissue **(B)**. EpCAM and CHGA gating were determined using EpCAM Fluorescence Minus One (FMO) controls and CHGA FMO controls for ileum tissue **(C)** and nasal tissue **(D)**. EpCAM (blue) and CHGA (red) expression shown as histograms compared to their respective FMO (grey) for ileum tissue **(E)** and nasal tissue **(F)**. FVD: Fixable Viability Dye eFluor™; EpPCAM: epithelial cellular adhesion molecule; CHGA: chromogranin A; AR = allergic rhinitis; CRSwNP = chronic rhinosinusitis with nasal polyps

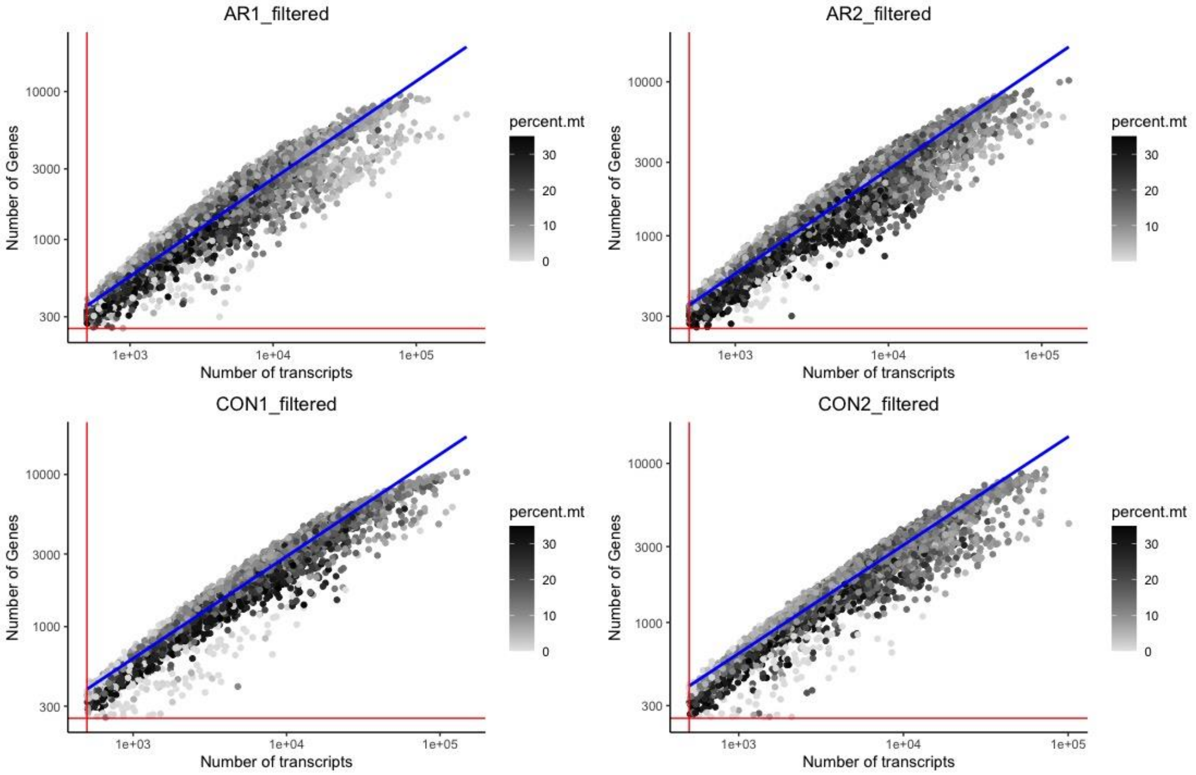

Supplementary figure 2: Quality control of the single cell RNA-sequencing dataset. Graphs showing the quality of each of the samples after filtering the datasets using the following parameters: number of genes > 250, number of transcripts > 500 and percentage of mitochondrial DNA < 35%.

AR1 = allergic rhinitis patient 1; AR2 = allergic rhinitis patient 2; CON1 = control subject 1; CON2 = control subject 2

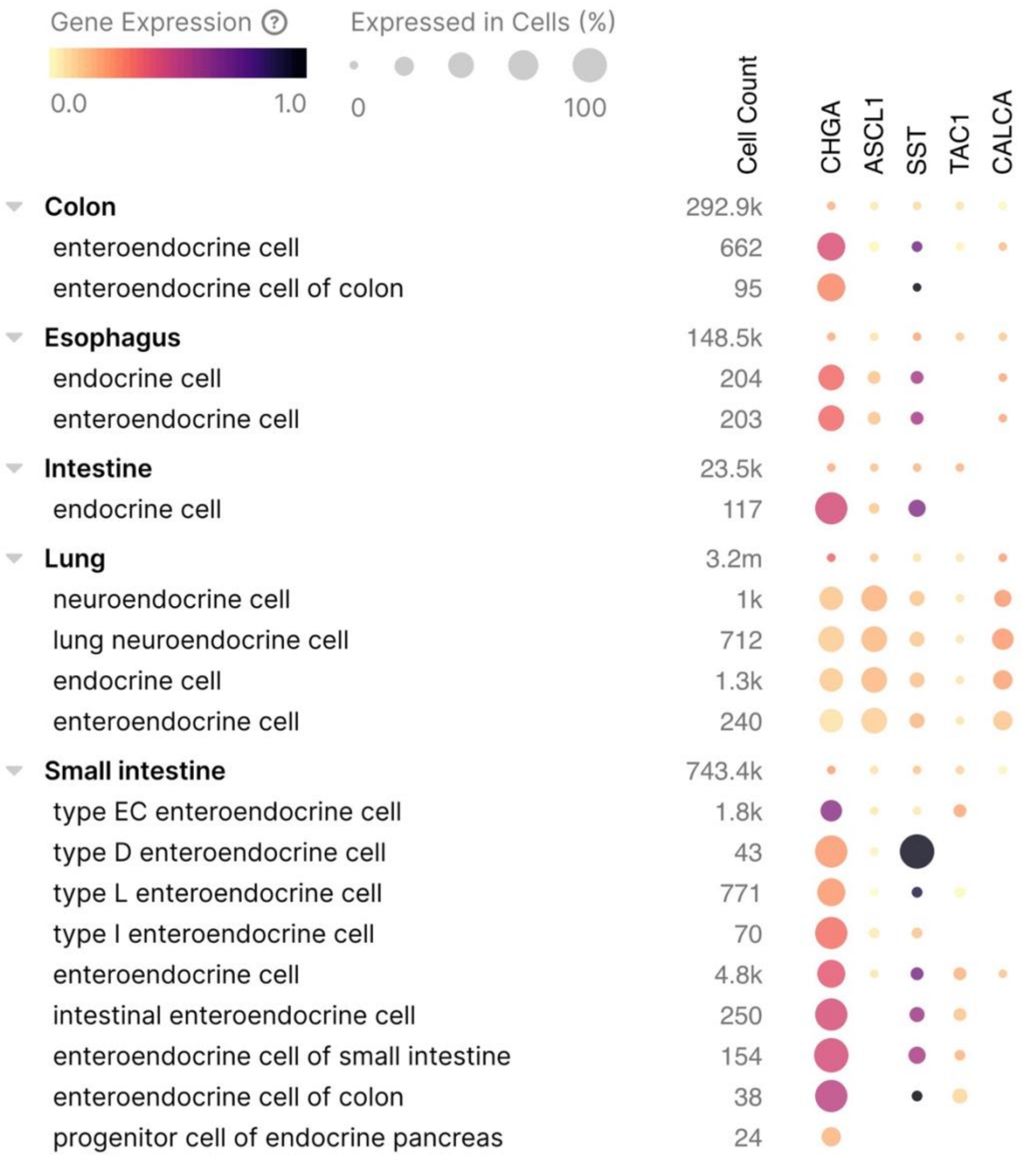

Supplementary figure 3: Dotplot of NEC markers in other human tissue based on the CZ CELLxGENE Discover database

Dotplot showing RNA expression levels of NEC markers *CHGA*, *ASLC1*, *SST*, *TAC1* and *CALCA* in human colon, human esophagus, human intestine, human lung and human small intestine tissue. Data and dotplot obtained from CZI Single-Cell Biology, BioRxiv 2023 (42).

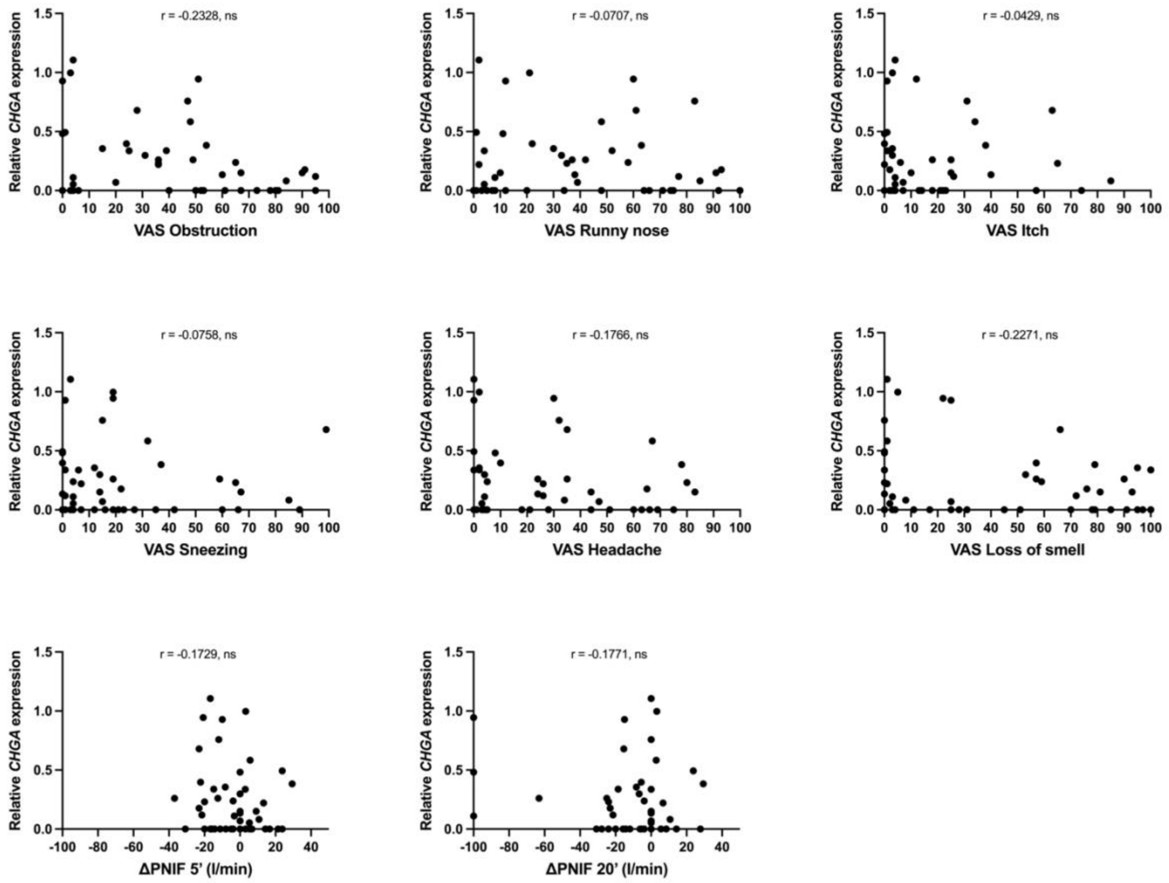

Supplementary figure 4: Correlation between the expression of the NEC marker *CHGA* and disease severity and nasal hyperreactivity

Correlation of *CHGA* mRNA expression with the VAS scores for obstruction, VAS score for runny nose, VAS score for itch, VAS score for sneezing, VAS score for headache, VAS score for loss of smell, change in PNIF 5 minutes after cold dry air provocation and change in PNIF 20 minutes after cold dry air provocation as measures of disease and NHR severity.

CHGA: chromogranin A; VAS = visual analogue score; NHR = nasal hyperreactivity; PNIF = peak nasal inspiratory flow; r = spearman r correlation value, ns = $p > 0.05$

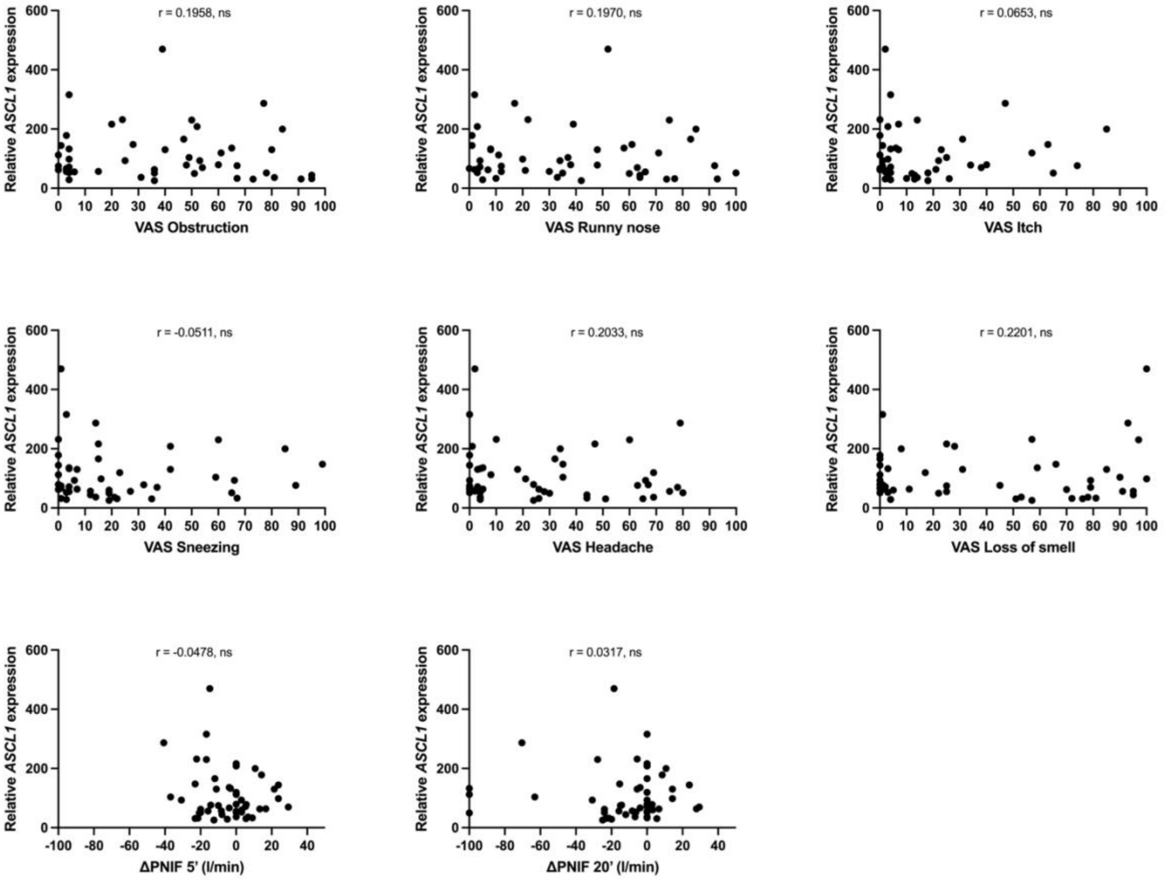

Supplementary figure 5: Correlation between the expression of the NEC marker *ASCL1* and disease severity and nasal hyperreactivity

Correlation of *ASCL1* mRNA expression with the VAS scores for obstruction, VAS score for runny nose, VAS score for itch, VAS score for sneezing, VAS score for headache, VAS score for loss of smell, change in PNIF 5 minutes after cold dry air provocation and change in PNIF 20 minutes after cold dry air provocation as measures of disease and NHR severity.

ASCL1 = Achaete-Scute Family bHLH transcription factor 1; VAS = visual analogue score; NHR = nasal hyperreactivity; PNIF = peak nasal inspiratory flow; r = spearman r correlation value, ns = $p > 0.05$

1.2 Supplementary Tables

Supplementary table 1: Patient's characteristics of isolated primary nasal epithelial cells

	**Control**	**AR**	**CRSwNP**	**p value**
**Total**	**6**	**6**	**6**	
Sex (M/F)	3/3	4/2	4/2	ns
Age	34 (18-70)	28 (20-32)	35 (27-75)	ns
Current smoker	0	0	0	ns
Asthma/COPD	0	1 (17%)	1 (17%)	ns

M = male, F = female, COPD = chronic obstructive pulmonary disease

Supplementary table 2: Patient's characteristics of nasal biopsies

	**Control**	**AR**	**CRSwNP**	**p value**
**Total**	**12**	**16**	**22**	
Sex (M/F)	7/5	9/7	16/6	ns
Age	36.5 (18-61)	31 (20-62)	50.5 (29-65)	AR vs CRSwNP: 0.0137
Current smoker	0 (0%)	5 (31%)	11 (50%)	0.0115
Asthma/COPD	0 (0%)	1 (6%)	3 (14%)	ns
Previous FESS	0 (0%)	1 (6%)	14 (64%)	<0,0001
Inferior conchotomy	0 (0%)	1 (6%)	3 (14%)	ns
VAS total sinonasal symptoms	0.3 ± 0.2	5.7 ± 2.0	3.8 ± 2.4	Control vs AR: <0.0001<br>Control vs CRSwNP: 0.0003
VAS obstructive	0.5 ± 0.7	6.1 ± 1.7	4.1 ± 3.2	Control vs AR: <0.0001<br>Control vs CRSwNP: 0.0028
VAS Running nose	0.6 ± 0.6	5.4 ± 3.0	4.3 ± 2.7	Control vs AR: <0.0001<br>Control vs CRSwNP: 0.0005
VAS Itch	0.2 ± 0.2	3.0 ± 2.4	1.6 ± 1.9	Control vs AR: 0,0001<br>Control vs CRSwNP: 0,0430
VAS Sneezing	0.4 ± 0.5	3.1 ± 3.0	2.6 ± 2.5	Control vs AR: 0.0016<br>Control vs CRSwNP: 0.0022
VAS headache	0.2 ± 0.3	3.7 ± 2.5	3.7 ± 3.0	Control vs AR: 0.0007<br>Control vs CRSwNP: 0.0004
VAS Smell	0.7 ± 1.9	3.8 ± 3.5	6.3 ± 3.3	Control vs CRSwNP: <0.0001
Nasal Hyperreactivity	0 (0%)	8 (50%)	9 (41%)	0.0108
ΔPNIF	-1.0 ± 12.3	-10.9 ± 14.9	-13.4 ± 17.1	ns

Exclusion criteria: use of any nasal medication, corticosteroids, antihistamines or saline lavages 1 week prior to the visit, prior use of allergen immunotherapy, relevant anatomical abnormalities in the nose, acute upper airway infection in the past 2 weeks, alcohol consumption in the past 24 hours, use of tricyclic antidepressants, intranasal drug-abuse in the past 12 months, pregnancy or breastfeeding and active malignancy

M = male, F = female, COPD = chronic obstructive pulmonary disease; FESS = functional endoscopic surgery; VAS = visual analogue scale; PNIF = peak nasal inspiratory flow

Supplementary table 3: Patient’s characteristics of ileum biopsies

	**Ileum**
**Total**	**5**
CD/CRC	4/1
Sex (M/F)	0/5
Age	73 (54-96)
Current smoker	1
Asthma/COPD	0
Corticosteroid use	1

CD= Crohn’s disease, CRC = colorectal cancer, M = male, F = female, COPD = chronic obstructive pulmonary disease

Supplementary table 4: Primer sequences for RT-qPCR

**Target Gene**	**Forward primer**	**Reverse primer**	**Hydrolysis probe**
CHGA	gggataccgaggtgatgaaatg	tcagaatggaaaggatccgttc	cgttgaggtcatctccgacacactttcc
ASCL1	agcaggagcttctcgacttcac	cgatcaccctgcttccaaag	ctggtcaggccctggtgcgaat
ACTB	ggacatccgcaaagacctgt	ctcaggaggagcaatgatcttgat	ctggcggcaccaccatgtaccct
RACK1	cactgtccaggatgagagcca	catacccttgaccagcttgtccc	tccgcttctcgcccaacagcag

CHGA = chromogranin A, *ASCL1* = Achaete-Scute Family BHLH Transcription Factor 1, *ACTB* = actin beta, *RACK1* = Receptor For Activated C Kinase 1
